# Assessing the Impact of Radiographic Realignment on Adult Spinal Deformity Patients with Sacroiliac Joint Pain at Presentation

**DOI:** 10.3390/jcm13123543

**Published:** 2024-06-17

**Authors:** Oluwatobi O. Onafowokan, Peter Tretiakov, Nathan Lorentz, Matthew Galetta, Ankita Das, Jamshaid Mir, Timothy Roberts, Peter G. Passias

**Affiliations:** Division of Spinal Surgery, Departments of Orthopaedic and Neurosurgery, NYU Langone Medical Center, New York Spine Institute, New York, NY 10003, USA

**Keywords:** adult spinal deformity, sacroiliac joint pain, realignment

## Abstract

**Background:** Adult spinal deformity (ASD) patients with concurrent sacroiliac joint (SIJ) pain are susceptible to worse postoperative outcomes. There is scarce literature on the impact of ASD realignment surgery on SIJ pain. **Methods**: Patients undergoing ASD realignment surgery were included and stratified by the presence of SIJ pain at the baseline (SIJP+) or SIJ pain absence (SIJP−). Mean comparison tests via ANOVA were used to assess baseline differences between both cohorts. Multivariable regression analyses analyzed factors associated with SIJ pain resolution/persistence, factoring in BMI, frailty, disability, and deformity. **Results**: A total of 464 patients were included, with 30.8% forming the SIJP+ cohort. At the baseline (BL), SIJP+ had worse disability scores, more severe deformity, higher BMI, higher frailty scores, and an increased magnitude of lower limb compensation. SIJP+ patients had higher mechanical complication (14.7 vs. 8.2%, *p* = 0.024) and reoperation rates (32.4 vs. 20.2%, *p* = 0.011) at 2 years. SIJP+ patients who subsequently underwent SI fusion achieved disability score outcomes similar to those of their SIJ− counterparts. Multivariable regression analysis revealed that SIJP+ patients who were aligned in the GAP lordosis distribution index were more likely to report symptom resolution at six weeks (OR 1.56, 95% CI: 1.02–2.37, *p* = 0.039), 1 year (OR 3.21, 2.49–5.33), and 2 years (OR 3.43, 2.41–7.12). SIJP− patients who did not report symptom resolution by 1 year and 2 years were more likely to demonstrate PI-LL > 5° (OR 1.36, 1.07–2.39, *p* = 0.045) and SVA > 20 mm (OR 1.62, 1.24–1.71 *p* = 0.017). **Conclusions**: SIJ pain in ASD patients may result in worsened pain and disability at presentation. Symptom resolution may be achieved in affected patients by adequate postoperative lumbar lordosis restoration.

## 1. Introduction

Adult spinal deformity (ASD) is a complex condition characterized by abnormal alignment and curvature of the spine in adult individuals [1]. It encompasses a range of spinal pathologies, including degenerative scoliosis, degenerative kyphosis, and spinal stenosis, among others. Surgical intervention is often required to correct the deformity and alleviate symptoms. Although surgical realignment can provide significant benefits, it is crucial to consider potential concurrent diagnoses and their impacts on patient outcomes [2,3].

One such diagnosis is sacroiliac joint (SIJ) pain or dysfunction, which may precede or even arise following ASD surgery [4]. The disruption of the normal biomechanics of these joints can lead to significant discomfort and functional impairment [5]. Despite the relevance of SIJ pain and its potential impact on postoperative outcomes, there is a paucity of research exploring the effects of surgical realignment on pre-existing SIJ pain. Understanding the relationship between radiographic parameters in ASD corrective surgery and SIJ pain could guide surgeons in optimizing their preoperative plans [6]. Given the intimate relationship between the sacroiliac joint and the lumbosacral spine, we suspect that restoration of lumbar lordosis and sagittal balance would be important in addressing SIJ pain.

Shin et al. conducted a retrospective study on the relationship between lumbopelvic sagittal alignment and SIJ pain following posterior lumbar interbody fusion and found that lumbopelvic sagittal imbalance may indeed play a role in the development of postoperative SIJ pain [7]. To the best of our knowledge, no other study has explored the relationship between radiographic alignment and SIJ pain with regard to patient-reported outcomes. The current study aims to investigate associations between SIJ pain and radiographic and clinical outcomes following ASD realignment surgery.

## 2. Materials and Methods

### 2.1. Data Source and Study Design

This was a retrospective analysis of a prospectively collected database of adult spinal deformity (ASD) patients. The inclusion criteria of this database have been detailed in the previous literature investigating the assessment and management of ASD [8,9,10]. Patients were included in the study if they were undergoing surgical intervention for ASD, had a lumbar primary sagittal plane deformity (as described by the Scoliosis Research Society’s spinal deformity classification), and had at least one of the following radiographic deformity parameters: sagittal vertical axis (SVA) > 5 cm, Cobb angle > 20°, pelvic tilt (PT) > 25°, or thoracic kyphosis (TK) > 60°. Patients were excluded if they had undergone prior lower limb joint replacement surgery. Institutional Review Board (IRB) approval was obtained prior to enrollment, and all the patients provided informed consent. The study was conducted in accordance with the Declaration of Helsinki and approved by the Ethics Committee of NYU School of Medicine (protocol code S13-00422; date of approval: 3/8/2013).

### 2.2. Data Collection and Radiographic Assessment

Baseline (BL) demographics, surgical details, and outcome data up to two years (2Y) following surgery were included. Demographic data collected included age and gender.

Frailty was determined using the ASD-modified frailty index [10,11]. Outcome data also involved health-related quality-of-life (HRQL) measurements, including the Oswestry disability index (ODI), and the Scoliosis Research Society’s 22-item score (SRS-22). The ODI is a scoring system for disability due to lumbar spine pathology. It is scored from 0 (no disability) to 100 (crippling disability). The SRS-22 is another scoring system, which quantifies the impacts of spinal deformity in 5 domains (pain, function, mental health, self-image, and satisfaction). It is scored from 1 (worst) to 5 (best quality of life).

Patients were stratified by the presence of sacroiliac joint pain or dysfunction (SIJP+) at the initial presentation or their absence (SIJP−). Sacroiliac joint (SIJ) pain or dysfunction was diagnosed by the presentation of at least 3 of Laslett et al.’s 5 provocative tests for SIJ pathology [12]. Patients were further stratified by age-adjusted criteria as being undercorrected, overcorrected, or matched [13].

Lateral spine radiographs were used to assess radiographic parameters at the baseline and follow-up intervals. EOS imaging incorporates both sagittal and coronal comprehensive views from the top of the skull to the bottom of the foot, with radiographic sagittal and coronal measurement and analysis supported by Spineview^®^ software 2.0 (Laboratory of Biomechanics, Paris, France) and Surgimap^®^ software (New York, NY, USA) [14,15,16]. The global alignment and proportion metrics were determined for included patients, as described by Yilgor et al. [17].

### 2.3. Statistical Analysis

Mean comparison analyses (T-tests, ANCOVA, and chi-squared tests) were used to assess differences between SIJP+ and SIJP− groups with regard to demographic, radiographic, and HRQL measures at the baseline and at all the follow-up timepoints. Backstep logistic regression modeling was used to assess factors predictive of the resolution of symptoms at the follow-up, adjusting for similar covariates in deformity, disability, and demographic factors. All the statistical analyses were performed using SPSS statistics software version 28 (IBM, Armonk, NY, USA).

## 3. Results

### 3.1. Cohort Overview

A total of 464 patients were included. The mean age and BMI were 63.4 ± 14.7 years and 29.1 ± 6.0 kg/m^2^, respectively. A total of 59% of the patients were female. At the baseline, 143 patients (30.8%) reported some degree of sacroiliac joint pain and formed the SIJP+ group. At the baseline, SIJP+ patients had higher BMIs (28.1 vs. 25 kg/m^2^, *p* = 0.009) and were frailer than SIJP− patients, as determined by the ASD-mFI (4.07 vs. 1.96, *p* < 0.001).

### 3.2. Baseline Radiographic and Disability Comparison

At the baseline, SIJP+ patients had worse overall deformity, as seen in pelvic incidence–lumbar lordosis (PI-LL) mismatch (22.5 vs. 9.1°, *p* = 0.033), and a worse sagittal vertical axis (SVA, 97.2 vs. 61.7 mm, *p* = 0.003) than SIJP− patients. SIJP+ patients also demonstrated greater compensation in the lower limbs, as measured by the knee angle (11 vs. 3.3°, *p* = 0.002) and ankle angle (7.1 vs. 3.8°, *p* = 0.004), than SIJP− patients.

With regard to HRQL metrics at the baseline, SIJP+ had worse ODI scores (49.7 vs. 30.5, *p* < 0.001) and SRS scores (2.7 vs. 3.3, *p* < 0.001) than SIJP−. The comorbidity burden and osteoporosis incidence were similar between the SIJP+ and SIJP− groups (Table 1).

### 3.3. Surgical Characteristics

Both groups were comparable in the number of levels fused anteriorly and posteriorly, rates of any osteotomy, and rates of 3-column osteotomies (15.1 vs. 16.3%, *p* = 0.354, Table 2). SIJP+ had longer operations (477.3 vs. 395.1 min, *p* = 0.037) and higher estimated blood losses (1489.4 vs. 1143.8 mL, *p* = 0.043) than SIJP−. There were no differences in the lengths of hospital stays. A total of 36.5% of the SIJP+ underwent concurrent SI fusion at the index operation compared to SIJP− (22.1%, *p* = 0.012).

### 3.4. Postoperative Outcomes

**Figure 1:**
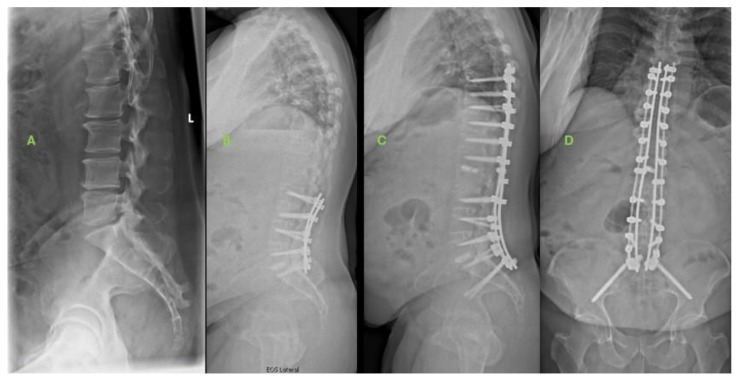
Example of SIJP+ patient. (**A**) Baseline radiographs of 59-year-old male with preoperative diagnoses of spinal stenosis, spondylosis, loss of lumbar lordosis, bilateral radiculopathy, and mild sacroiliac joint pain. (**B**) Six-week postoperative radiographs. The patient underwent L2-S1 anterior lumbar interbody fusion and L2-S1 posterior spinal fusion. Symptoms initially improved, but the patient developed progressive SIJ and back pain due to adjacent-segment pathology with proximal junctional kyphosis and thoracic stenosis and myelopathy. The patient subsequently underwent T10-L2 posterior spinal fusion with SIJ fusion 17 months after the index surgery. (**C**,**D**) Anteroposterior and lateral radiographs at 2 years post index surgery. SIJ symptoms resolved, with improvement in disability but still some residual activity-dependent back pain.

**Figure 2:**
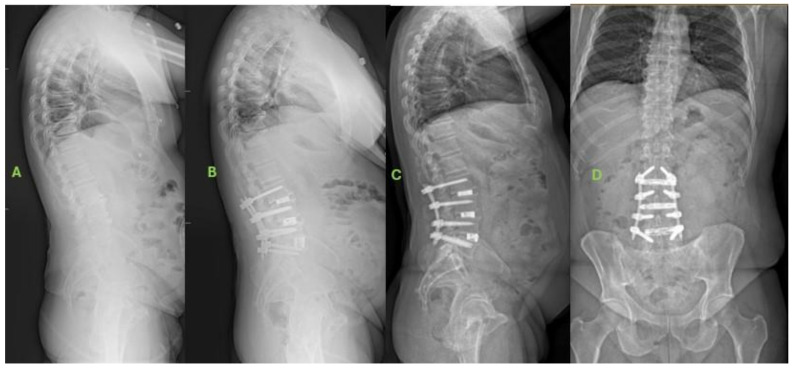
Example of SIJP− patient. (**A**) Baseline radiographs of 65-year-old male with preoperative diagnoses of loss of lumbar lordosis, lumbar spondylosis, bilateral radiculopathy, and spinal stenosis. (**B**) Six-week postoperative radiographs. The patient underwent L2-L5 lateral lumbar interbody fusion and L2-S1 posterior spinal fusion. (**C**,**D**) Two-year anteroposterior and lateral radiographs. Significant improvement in preoperative symptoms with maintained alignment still demonstrated at 2 years.

Figure 1 and Figure 2 illustrate case examples of a patient from each cohort. At six weeks postoperatively, although there were no differences between both groups in spinopelvic parameters, SIJP+ exhibited worse ODI scores (45.5 vs. 27.6, *p* = 0.009) than SIJP−. SIJP+ patients did experience significant ODI improvements between the baseline and six weeks postoperatively (49.7 vs. 45.5, *p* = 0.048). SIJP+ patients also demonstrated greater residual knee and ankle compensation than SIJP− (Table 2).

SIJP+ still demonstrated inferior ODI scores at 1 year compared to SIJP−. However, this difference had subsided by 2 years (Table 3). There were no differences in spinopelvic and lower limb radiographic parameters at 1 and 2 years. By 2 years, SIJP+ had experienced more mechanical complications and reoperations than SIJP−. A total of 46 SIJP+ patients (32.2%) and 65 SIJP− patients (20.2%) underwent reoperations for various reasons. Of the SIJP+ patients undergoing reoperation, 30% underwent subsequent sacroiliac joint (SIJ) fusion for persistent SIJ pain/dysfunction, 68% underwent reoperation for pseudoarthrosis, 21% had proximal junctional failure (PJF), 10% had rod breakage, 14% had interbody device dislodgment, and 11% had screw loosening/pull-out. In the SIJP− cohort undergoing reoperation, 63% of the patients underwent reoperation for pseudoarthrosis, 16% had PJF, 13% had rod breakage, 9% had interbody device dislodgment, and 12% had screw loosening/pull-out. A sub-analysis of the SIJP+ patients undergoing subsequent SIJ fusion revealed no difference in the ODI at 2 years when comparing them to SIJP− patients (20.5 vs. 18.2, *p* = 0.236).

In terms of the SIJ symptom resolution in SIJP+, 9.8% reported the resolution of symptoms by 6W, with 47.4% at 1 year and 65% at two years postoperatively. Of note, the SIJP+ patients undergoing subsequent SIJ fusion reported the complete resolution of SIJ symptoms by two years. These patients underwent SIJ fusion at a median of 14 months after the primary procedure. In a logistic regression analysis controlling for the BMI, frailty, baseline deformity (PI-LL and SVA), disability (ODI and SRS), and sacroiliac joint fusion, SIJP+ patients who were aligned in the GAP lordosis distribution index were more likely to report symptom resolution at six weeks (OR 1.56, 95% CI: 1.02–2.37, *p* = 0.039), 1 year (OR 3.21, 2.49–5.33), and 2 years (OR 3.43, 2.41–7.12). SIJP+ patients who did not report symptom resolution by 1 year and 2 years were more likely to demonstrate PI-LL > 5° (OR 1.36, 1.07–2.39, *p* = 0.045) and SVA > 20 mm (OR 1.62, 1.24–1.71 *p* = 0.017). 

## 4. Discussion

Sacroiliac joint (SIJ) pain is a common and debilitating condition often associated with spinal abnormalities, leading to significant impairment in the quality of life [18]. Although the benefits of surgical correction for adult spinal deformities are well established, limited evidence exists regarding the effects of ASD correction on sacroiliac joint pain. This study aimed to investigate the relationship between patients with SIJ pain and patient-reported outcomes, as well as the effect of surgical realignment on sacroiliac pain.

SIJ injury has been proposed to be the result of combined axial loading and sudden rotatory forces, eventually resulting in SIJ pain [19]. These forces influence a spectrum of pathological alterations to different peri-SIJ structures, including micro- or macro-fractures, capsular and ligamentous tensions, inflammatory changes, and structural hypo- or hypermobility [20]. Other influential factors on SIJ pain include lower limb length discrepancy (LLD) and prior lumbar fusion. LLD may contribute to SIJ pain because of the imbalance in the mechanical alignment of the SIJs, resulting in an unequal load distribution across the SIJs. A study by Kiapour et al. demonstrated that as LLD increased from 1 to 3 cm, the SIJ stresses across both SIJs increased accordingly, with the stress most significant on the SIJ of the longer leg [21]. Prior lumbar fusion can influence SIJ pain by increasing the angular motion and stress across the SIJs and by adjacent-segment degeneration following fusions with lower instrumented vertebra around L5/S1 [22,23]. Further factors associated with SIJ pain may include excessive physical exercise, gait abnormalities, obesity, connective tissue disorders, pregnancy, infection, and spondyloarthropathies [24].

Patients with preoperative SIJ pain in this study demonstrated worse sagittal malalignment at the baseline, as denoted by PI-LL and SVA. Sagittal PI-LL mismatch is recognized as an important parameter that reflects the spinal alignment and balance [25]. Higher PI-LL values are associated with an elevated risk for adjacent-segment degeneration and the need for revision surgery [26,27]. As previously cited, Shin et al. conducted a similar study addressing the relationship between lumbopelvic sagittal alignment and SI pain after posterior lumbar interbody fusion (PLIF) surgery [7]. Although their study only examined the effect of correcting PI-LL mismatch on postoperative SIJ pain, they found a strongly positive correlation between PI-LL values below 10 degrees and a decrease in patient-reported SIJ pain [7]. Similarly, in our study, patients who had higher PI-LL values at 1- and 2-year follow-ups were less likely to report symptom resolution.

Cho et al. conducted a retrospective study comparing patients who underwent lumbar fusion and developed subsequent SIJ pain with those who did not develop postoperative SIJ pain. They found that patients with SIJ pain exhibited pelvic retroversion and a greater pelvic tilt (PT) [28]. Pelvic retroversion is a notable compensatory mechanism for sagittal malalignment. Although the patients, in our study, with SIJ pain did not demonstrate significantly higher PTs, these patients demonstrated a greater compensatory magnitude in the lower limbs at the knee and ankle. This indicates that concurrent SIJ pain may cause significant sagittal malalignment exacerbations if affected patients are unable to recruit compensatory mechanisms.

Regarding HRQL metrics, a study by Diesing et al. investigated the relationship between radiographic parameters and persistent sacroiliac joint syndrome following ASD corrective surgery and found that patients with persistent SIJ pain had significantly reduced improvements in HRQL outcomes compared to patients without persistent SIJ pain and that persistent SIJ pain following surgery may be avoided with the restoration of the lumbosacral sagittal alignment [29]. Our findings are also in line with this, as SIJP+ patients demonstrated inferior ODI scores at all the timepoints. Notably, SIJP+ patients who subsequently underwent SIJ fusion demonstrated complete symptom resolution. This indicates that it is worth considering SIJ fusion concurrent with realignment surgery to improve outcomes in patients with preoperative SIJ pain.

Our study is strengthened by the sample size as well as the investigation of an understudied aspect of adult spinal deformities. It is also strengthened by the prospective enrolment of patients and standardization of perioperative care. We also acknowledge limitations to the present study. We utilized a retrospective design, which may introduce inherent biases, including potential selection bias. The retrospective nature of the study limits our ability to control all the potential confounding variables and ascertain causality. Additionally, reliance on medical records for data collection may introduce inaccuracies or missing information, leading to potential information bias. This study is also based on a single surgeon series, which may introduce biases related to surgeon expertise, techniques, and patient selection, which may limit the ability of the study to be generalized. Furthermore, we did not include data or analyses on morphological SIJ pathology, such as degenerative change or erosion, and we recognize that these conditions may also potentially impact patient outcomes. We also acknowledge that the impact of the SIJ pathology on patient outcomes may last over a much longer period than the 2-year study length we observed. Future studies with significantly longer follow-ups will be invaluable for enhancing the findings demonstrated in the present study.

## 5. Conclusions

Adult spinal deformity patients with concomitant SIJ pain self-report significantly greater pain and disability scores at presentation than those without SIJ symptoms. In patients undergoing corrective ASD fusion, almost 50% of the patients with preoperative SI joint pain experienced a resolution of SI joint symptoms by one-year postoperatively. Postoperative lordosis restoration was found to be predictive of improvement in SIJ pain in affected patients by two years.

## Figures and Tables

**Table 1:** Baseline comparisons between cohorts.

	SIJP+	SIJP−	Sig.
Age, years	63.4 ± 12.7	58.3 ± 19.4	0.389
BMI, kg/m^2^	28.1 ± 5.5	25 ± 5	0.009
CCI	1.37 ± 0.48	1.85 ± 0.14	0.093
ASD-mFI	4.07 ± 3.27	1.96 ± 3.51	<0.001
Osteoporosis, %	18	21	0.945
ODI	49.7 ± 15.7	30.5 ± 17.3	<0.001
SRS-22	2.7 ± 0.54	3.3 ± 0.59	<0.001
SVA, mm	97.2 ± 67.3	61.7 ± 67.4	0.002
TK, °	−39.7 ± 19.2	−42.7 ± 24	0.543
PT, °	27.3 ± 10.2	22.5 ± 11.5	0.126
LL,	31.3 ± 22	45.1 ± 26.6	0.023
PI-LL, °	22.5 ± 20.7	9.1 ± 25.5	0.033
T1PA, °	29.5 ± 12.8	21.9 ± 13.8	0.051
SFA, °	203.3 ± 9.9	202.4 ± 10.8	0.736
KA, °	11 ± 9.9	3.3 ± 8.6	0.002
AA, °	7.1 ± 4.8	3.8 ± 4.5	0.004
GAP score	9.1 ± 2.3	7.4 ± 2	0.037

AA = ankle angle; ASD-mFI = adult spinal deformity modified frailty index; BMI = body mass index; CCI = Charlson comorbidity index; GAP = global alignment and proportion score; KA = knee angle; LL = lumbar lordosis; PT = pelvic tilt; PI-LL = pelvic incidence–lumbar lordosis mismatch; ODI = Oswestry disability index; SRS-22 = Scoliosis Research Society’s 22-item score; SFA = sacrofemoral angle; Sig. = significance (*p*-value); SVA = sagittal vertical axis; TK = thoracic kyphosis; T1PA = T1-pelvic angle.

**Table 2:** Surgical factor and perioperative outcome comparisons.

	SIJP+	SIJP−	Sig.
EBL, mL	1489.4 ± 1126.8	1143.8 ± 1023.3	0.043
Operative time, mins	477.3 ± 171.1	428.9 ± 197.5	0.037
LOS, days	6.9 ± 3.7	6.9 ± 3.9	0.934
Levels fused (posterior)	10.4 ± 3.8	9.6 ± 4.3	0.053
Levels fused (anterior)	2.3 ± 0.8	2.2 ± 1.1	0.973
Interbody fusion levels	3.1 ± 3.3	2.9 ± 3.2	0.768
6W ODI	45.5 ± 18.7	27.6 ± 18.6	0.009
6W SRS-22	3.2 ± 0.58	3.5 ± 0.57	0.069
6W SVA, mm	18.8 ± 41.4	9.9 ± 38.3	0.021
6W PI-LL, °	3.3 ± 13	−0.2 ± 10.8	0.319
6W T1PA, °	16.2 ± 9.5	14.6 ± 8.8	0.541
6W PT, °	21 ± 9.9	20.3 ± 9	0.807
6W TK, °	−44 ± 13.7	−43.6 ± 13.7	0.855
6W SFA, °	201.5 ± 10	204.3 ± 8.3	0.329
6W KA, °	5.7 ± 8	−0.7 ± 7.4	0.003
6W AA, °	6.1 ± 4.6	3.1 ± 4.2	0.036

EBL = estimated blood loss; LOS = length of hospital stay; ODI = Oswestry disability index; Sig. = significance (*p*-value); PI-LL—pelvic incidence lumbar lordosis mismatch; SRS-22 = Scoliosis Research Society’s 22-item score; T1PA = T1-pelvic angle; 6W = 6 weeks.

**Table 3:** Postoperative comparisons.

	SIJP+	SIJP−	Sig.
Y1 ODI	27.3 ± 18.6	19 ± 15.6	0.045
Y2 ODI	28.7 ± 19.2	18.2 ± 18.8	0.036
Y1 SRS-22	3.8 ± 0.7	3.9 ± 0.6	0.567
Y2 SRS-22	3.7 ± 0.5	3.7 ± 0.3	0.585
Y1 PI-LL, °	1 ± 12.3	1.9 ± 13.9	0.571
Y2 PI-LL, °	1.8 ± 10.5	1.2 ± 10.6	0.698
Y1 SVA, mm	21.4 ± 41.9	20.3 ± 43.1	0.680
Y2 SVA, mm	19.6 ± 39.4	20 ± 37.2	0.432
Y1 T1PA, °	15.2 ± 9.7	16.6 ± 11.9	0.387
Y2 T1PA, °	15 ± 9.8	14.3 ± 10.4	0.215
Y1 PT, °	19.4 ± 10.2	22.6 ± 11.4	0.366
Y2 PT, °	21.3 ± 9.7	22.1 ± 9.6	0.451
Y1 SFA, °	204.3 ± 10.5	201.3 ± 8.9	0.491
Y2 SFA, °	203 ± 8.6	202.6 ± 8.5	0.361
Y1 KA, °	4.1 ± 5.1	2.8 ± 6.9	0.476
Y2 KA, °	4.4 ± 4.5	2.7 ± 5.8	0.334
Y1 AA, °	5.8 ± 3.7	4.6 ± 4	0.514
Y2 AA, °	5.6 ± 2.8	4.3 ± 3.3	0.772
Mechanical complications, %	14.7	8.2	0.024
Reoperations, %	32.2	20.2	0.011
Y1 GAP score	6.1 ± 1.5	5.8 ± 1.3	0.353
Y2 GAP score	6 ± 0.9	5.9 ± 1	0.281

AA = ankle angle; GAP = global alignment and proportion score; KA = knee angle; LL = lumbar lordosis; PT = pelvic tilt; PI-LL = pelvic incidence–lumbar lordosis mismatch; ODI = Oswestry disability index; SRS-22 = Scoliosis Research Society’s 22-item score, SFA = sacrofemoral angle; Sig. = significance (*p*-value); SVA = sagittal vertical axis; T1PA = T1-Pelvic Angle; Y1 = Year 1; Y2 = Year 2.

## Data Availability

The data used in this study is not publicly available due to Health Insurance Portability and Accountability Act and Institutional restrictions.
